# Methyl 2-methyl-4-(oxiran-2-ylmeth­oxy)-2*H*-1,2-benzothia­zine-3-carboxyl­ate 1,1-dioxide

**DOI:** 10.1107/S1600536810000668

**Published:** 2010-01-09

**Authors:** Matloob Ahmad, Hamid Latif Siddiqui, Muhammad Zia-ur-Rehman, Mark R. J. Elsegood, George W. Weaver

**Affiliations:** a Institute of Chemistry, University of the Punjab, Lahore 54590, Pakistan; b Applied Chemistry Research Centre, PCSIR Laboratories Complex, Ferozpure Road, Lahore 54600, Pakistan; c Chemistry Department, Loughborough University, Loughborough, Leicestershire LE11 3TU, England

## Abstract

In the title compound, C_14_H_15_NO_6_S, the thia­zine ring adopts a distorted half-chair conformation. The structure displays several cooperative weak inter­molecular C—H⋯O hydrogen-bonding inter­actions, giving rise to a two-dimensional sheet packing motif. The CH_2_ group in the meth­oxy linker to the oxirane ring, and the CH group in that ring, exhibit twofold positional disorder. The three-membered oxirane ring is twisted approximately perpendicular with respect to thia­zine ring (dihedral angle = 60/86° for the major/minor disorder components). 1,2-Benzothia­zines of this kind have a wide range of biological activities and are mainly used as medicines in the treatment of inflammation and rheumatoid arthritis.

## Related literature

For the synthesis of related mol­ecules, see: Zia-ur-Rehman *et al.* (2006 ▶, 2007 ▶, 2009 ▶). For the biological activity of 1,2-benzothia­zine 1,1-dioxides, see: Bihovsky *et al.* (2004 ▶); Fabiola *et al.* (1998 ▶); Kojić-Prodić & Rużić-Toroš (1982 ▶). For similar mol­ecules, see: Ahmad *et al.* (2008 ▶); Arshad *et al.* (2009 ▶). For reference bond-length data, see: Weast *et al.* (1984 ▶).
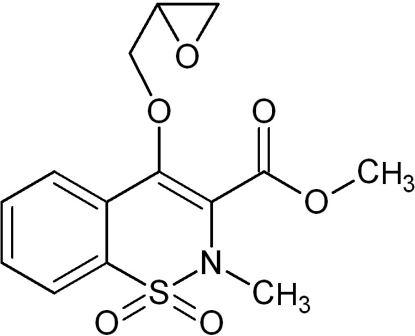


## Experimental

### 

#### Crystal data

C_14_H_15_NO_6_S
*M* *_r_* = 325.33
Monoclinic, 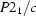

*a* = 7.2007 (3) Å
*b* = 12.8435 (6) Å
*c* = 15.7820 (7) Å
β = 96.5250 (7)°
*V* = 1450.10 (11) Å^3^
*Z* = 4
Mo *K*α radiation
μ = 0.25 mm^−1^
*T* = 300 K
0.44 × 0.37 × 0.24 mm

#### Data collection

Bruker APEXII CCD diffractometer
Absorption correction: multi-scan (*SADABS*; Sheldrick, 2004 ▶) *T*_min_ = 0.897, *T*_max_ = 0.942
11439 measured reflections
4516 independent reflections
3651 reflections with *I* > 2σ(*I*)
*R*_int_ = 0.016

#### Refinement

*R*[*F* ^2^ > 2σ(*F* ^2^)] = 0.051
*wR*(*F* ^2^) = 0.155
*S* = 1.08
4516 reflections
220 parameters
H-atom parameters constrained
Δρ_max_ = 0.70 e Å^−3^
Δρ_min_ = −0.27 e Å^−3^

### 

Data collection: *APEX2* (Bruker, 2007 ▶); cell refinement: *SAINT* (Bruker, 2007 ▶); data reduction: *SAINT*; program(s) used to solve structure: *SHELXS97* (Sheldrick, 2008 ▶); program(s) used to refine structure: *SHELXL97* (Sheldrick, 2008 ▶); molecular graphics: *SHELXTL* (Sheldrick, 2008 ▶); software used to prepare material for publication: *SHELXTL* and local programs.

## Supplementary Material

Crystal structure: contains datablocks I, global. DOI: 10.1107/S1600536810000668/bt5151sup1.cif

Structure factors: contains datablocks I. DOI: 10.1107/S1600536810000668/bt5151Isup2.hkl

Additional supplementary materials:  crystallographic information; 3D view; checkCIF report

## Figures and Tables

**Table 1:** Hydrogen-bond geometry (Å, °)

*D*—H⋯*A*	*D*—H	H⋯*A*	*D*⋯*A*	*D*—H⋯*A*
C7—H7⋯O6^i^	0.93	2.49	3.377 (3)	158
C15—H15⋯O3^ii^	0.98	2.50	3.317 (4)	140

Symmetry codes: (i) 
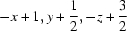
; (ii) 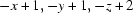.

**Fig. 1.**
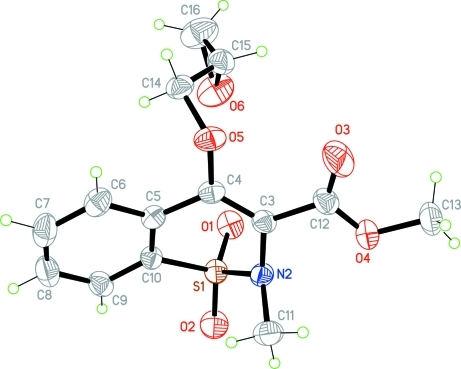
The molecular structure of (I), with displacement ellipsoids at the 40% probability level. The minor occupied site of the disordered atoms has been omitted.

**Fig. 2.**
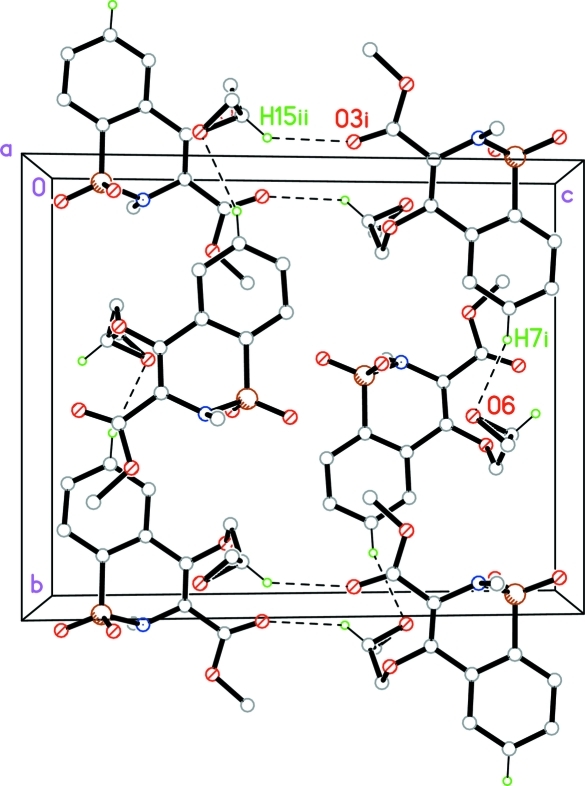
Perspective view of one thick layer of the crystal packing showing weak hydrogen-bonding interactions (dashed lines). H atoms not involved in hydrogen bonding have been omitted for clarity. i = 1 - x, y - 1/2, 1.5 - z; ii = x, 1/2 - y, z - 1/2

## supplementary crystallographic information

### Comment

Due to the verstaile applications of 1,2-benzothiazine 1,1-dioxides, much attention has been given to their synthesis. Some derivatives act as potent calpain I inhibitors (Bihovsky *et al.*, 2004) while others possess anti-bacterial, anti-fungal and anti-oxidant properties (Zia-ur-Rehman *et al.*, 2006, 2009). In continuation of our work on the synthesis (Zia-ur-Rehman *et al.*, 2006), biological activity (Zia-ur-Rehman *et al.*, 2009) and crystal structures (Zia-ur-Rehman *et al.*, 2007; Ahmad *et al.*, 2008; Arshad *et al.*, 2009) of various 1,2-benzothiazine-1,1-dioxides, we herein report the crystal structure of the title compound (I) (scheme and Fig. 1). Like the previously reported 1,2-benzothiazine1,1-dioxides (Zia-ur-Rehman *et al.*, 2007; Ahmad *et al.*, 2008; Arshad *et al.*, 2009), the thiazine ring involving two double bonds, exhibits a distorted half-chair conformation; with atoms S1/C10/C5/C4 coplanar within ±0.022 Å and N2 and C3 lying 0.961 and 0.525 Å respectively out of this plane. The geometry at N2 is pyramidal. The C10—S1 [1.7484 (17) Å] bond is shorter than a normal C—S single bond (1.81–2.55 Å) (Weast *et al.*, 1984) due to partial double bond character and this value is in agreement with similar, partially delocalized, bonds (Kojić-Prodić & Rużić-Toroš, 1982; Fabiola *et al.*, 1998]. The

positions the partially disordered oxirane group approximately perpendicular to the planar portion of the thiazine ring; dihedral angles between C4/C5/C10/S1 and the two diordered oxirane positions: 103 (major) and 108° (minor). There are two significant, intermolecular C—H···O interactions (Fig 2 & Table 1). Each molecule makes a total of four such interactions, two as donor and two as acceptor, resulting in a two-dimensional thick sheet structure, where the depth of the sheet is due to the elevation of the methoxy-oxirane group with respect to the thiazine ring system.

### Experimental

A mixture of methyl 4-hydroxy-2-methyl-2*H*-1,2-benzothiazine-3-carboxylate 1,1-dioxide (1.33 g, 5.0 mmol), 1-chloro-2,3-epoxypropane (2.313 g, 25.0 mmol), anhydrous potassium carbonate (10.0 g) and acetonitrile (100 ml) was stirred and refluxed for a period of 7 h. After removal of acetonitrile and excess 1-chloro-2,3-epoxypropane under vacuum, chloroform (30 ml) was added and the resultant mixture was filtered. The filtrate was washed with water to remove potassium carbonate, dried with anhydrous sodium sulfate and filtered. Slow evaporation of the solvent afforded the crystalline product.

### Refinement

H atoms were refined using a riding model with *U*_eq_ set to be 1.2 times that of the carrier atom (1.5 times for methyl H, and refined with rotational freedom). Atoms C14, C15, and that H atoms on C16 were refined over two sets of positions with major occupancy 64.8 (6)%.

### Crystal data

**Table:**

C_14_H_15_NO_6_S	*F*(000) = 680
*M**_r_* = 325.33	*D*_x_ = 1.490 Mg m^−^^3^
Monoclinic, *P*2_1_/*c*	Mo *K*α radiation, λ = 0.71073 Å
Hall symbol: -P 2ybc	Cell parameters from 4610 reflections
*a* = 7.2007 (3) Å	θ = 2.6–31.4°
*b* = 12.8435 (6) Å	µ = 0.25 mm^−^^1^
*c* = 15.7820 (7) Å	*T* = 300 K
β = 96.5250 (7)°	Block, colourless
*V* = 1450.10 (11) Å^3^	0.44 × 0.37 × 0.24 mm
*Z* = 4	

### Data collection

**Table:**

Bruker APEXII CCD diffractometer	4516 independent reflections
Radiation source: fine-focus sealed tube	3651 reflections with *I* > 2σ(*I*)
graphite	*R*_int_ = 0.016
ω rotation with narrow frames scans	θ_max_ = 31.7°, θ_min_ = 2.1°
Absorption correction: multi-scan (*SADABS*; Sheldrick, 2004)	*h* = −10→10
*T*_min_ = 0.897, *T*_max_ = 0.942	*k* = −18→14
11439 measured reflections	*l* = −22→17

### Refinement

**Table:**

Refinement on *F*^2^	Primary atom site location: structure-invariant direct methods
Least-squares matrix: full	Secondary atom site location: difference Fourier map
*R*[*F*^2^ > 2σ(*F*^2^)] = 0.051	Hydrogen site location: inferred from neighbouring sites
*wR*(*F*^2^) = 0.155	H-atom parameters constrained
*S* = 1.08	*w* = 1/[σ^2^(*F*_o_^2^) + (0.0788*P*)^2^ + 0.4338*P*] where *P* = (*F*_o_^2^ + 2*F*_c_^2^)/3
4516 reflections	(Δ/σ)_max_ = 0.001
220 parameters	Δρ_max_ = 0.70 e Å^−^^3^
0 restraints	Δρ_min_ = −0.26 e Å^−^^3^

### Special details

**Table:**

Geometry. All e.s.d.'s (except the e.s.d. in the dihedral angle between two l.s. planes) are estimated using the full covariance matrix. The cell e.s.d.'s are taken into account individually in the estimation of e.s.d.'s in distances, angles and torsion angles; correlations between e.s.d.'s in cell parameters are only used when they are defined by crystal symmetry. An approximate (isotropic) treatment of cell e.s.d.'s is used for estimating e.s.d.'s involving l.s. planes.
Refinement. Refinement of *F*^2^ against ALL reflections. The weighted *R*-factor *wR* and goodness of fit *S* are based on *F*^2^, conventional *R*-factors *R* are based on *F*, with *F* set to zero for negative *F*^2^. The threshold expression of *F*^2^ > σ(*F*^2^) is used only for calculating *R*-factors(gt) *etc*. and is not relevant to the choice of reflections for refinement. *R*-factors based on *F*^2^ are statistically about twice as large as those based on *F*, and *R*- factors based on ALL data will be even larger.

### Fractional atomic coordinates and isotropic or equivalent isotropic displacement parameters (Å^2^)

**Table:**

	*x*	*y*	*z*	*U*_iso_*/*U*_eq_	Occ. (<1)
S1	0.34508 (6)	0.47351 (3)	0.61034 (2)	0.04004 (14)	
O1	0.53323 (19)	0.44619 (11)	0.64070 (9)	0.0512 (3)	
O2	0.2622 (2)	0.43268 (12)	0.53097 (8)	0.0601 (4)	
N2	0.2133 (2)	0.44064 (11)	0.68409 (9)	0.0387 (3)	
C3	0.2671 (2)	0.48744 (13)	0.76543 (10)	0.0373 (3)	
C4	0.3247 (2)	0.58822 (13)	0.76958 (10)	0.0368 (3)	
C5	0.3205 (2)	0.65332 (13)	0.69280 (10)	0.0366 (3)	
C6	0.3032 (3)	0.76159 (14)	0.69774 (14)	0.0481 (4)	
H6	0.3046	0.7937	0.7506	0.058*	
C7	0.2842 (3)	0.82078 (16)	0.62423 (17)	0.0594 (5)	
H7	0.2724	0.8927	0.6283	0.071*	
C8	0.2823 (3)	0.77530 (17)	0.54444 (16)	0.0571 (5)	
H8	0.2684	0.8163	0.4956	0.069*	
C9	0.3014 (3)	0.66804 (16)	0.53801 (12)	0.0465 (4)	
H9	0.3006	0.6364	0.4850	0.056*	
C10	0.3215 (2)	0.60897 (13)	0.61187 (10)	0.0364 (3)	
C11	0.0099 (3)	0.43184 (18)	0.65850 (14)	0.0550 (5)	
H11A	−0.0408	0.4996	0.6443	0.082*	
H11B	−0.0493	0.4034	0.7048	0.082*	
H11C	−0.0121	0.3869	0.6098	0.082*	
C12	0.2533 (3)	0.42003 (16)	0.84103 (11)	0.0456 (4)	
O3	0.2713 (3)	0.44879 (15)	0.91356 (10)	0.0813 (6)	
O4	0.2175 (2)	0.32170 (12)	0.81730 (9)	0.0546 (3)	
C13	0.2062 (3)	0.2467 (2)	0.88466 (17)	0.0661 (6)	
H13A	0.1027	0.2636	0.9154	0.099*	
H13B	0.3199	0.2482	0.9229	0.099*	
H13C	0.1885	0.1783	0.8605	0.099*	
O5	0.37071 (18)	0.63669 (11)	0.84539 (8)	0.0469 (3)	
C14	0.5579 (4)	0.6871 (3)	0.8572 (2)	0.0451 (8)	0.646 (6)
H14A	0.5811	0.7217	0.8049	0.054*	0.646 (6)
H14B	0.5605	0.7392	0.9018	0.054*	0.646 (6)
C15	0.7068 (5)	0.6087 (3)	0.8807 (2)	0.0500 (8)	0.646 (6)
H15	0.6875	0.5604	0.9270	0.060*	0.646 (6)
C16	0.8933 (4)	0.6281 (2)	0.8656 (2)	0.0836 (8)	
H16A	0.9917	0.5941	0.9028	0.100*	0.646 (6)
H16B	0.9238	0.6977	0.8477	0.100*	0.646 (6)
H16C	0.9273	0.5999	0.9223	0.100*	0.354 (6)
H16D	0.9886	0.6711	0.8444	0.100*	0.354 (6)
O6	0.7826 (3)	0.56506 (15)	0.80605 (13)	0.0782 (5)	
C14X	0.5510 (8)	0.6227 (7)	0.8916 (4)	0.057 (2)	0.354 (6)
H14C	0.5688	0.5497	0.9059	0.069*	0.354 (6)
H14D	0.5578	0.6618	0.9444	0.069*	0.354 (6)
C15X	0.7008 (8)	0.6571 (6)	0.8428 (5)	0.0577 (19)	0.354 (6)
H15X	0.6761	0.7179	0.8057	0.069*	0.354 (6)

### Atomic displacement parameters (Å^2^)

**Table:**

	*U*^11^	*U*^22^	*U*^33^	*U*^12^	*U*^13^	*U*^23^
S1	0.0542 (3)	0.0363 (2)	0.0306 (2)	0.00076 (16)	0.00890 (16)	−0.00584 (14)
O1	0.0530 (7)	0.0515 (7)	0.0514 (8)	0.0115 (6)	0.0160 (6)	−0.0025 (6)
O2	0.0911 (11)	0.0555 (8)	0.0335 (6)	−0.0064 (7)	0.0059 (7)	−0.0133 (6)
N2	0.0468 (7)	0.0380 (7)	0.0313 (6)	−0.0071 (6)	0.0045 (5)	−0.0030 (5)
C3	0.0402 (8)	0.0430 (8)	0.0289 (7)	−0.0018 (6)	0.0050 (6)	−0.0006 (6)
C4	0.0378 (7)	0.0428 (8)	0.0304 (7)	−0.0006 (6)	0.0065 (5)	−0.0093 (6)
C5	0.0362 (7)	0.0358 (7)	0.0384 (8)	−0.0005 (6)	0.0070 (6)	−0.0037 (6)
C6	0.0459 (9)	0.0390 (8)	0.0603 (11)	0.0014 (7)	0.0103 (8)	−0.0083 (8)
C7	0.0552 (11)	0.0375 (9)	0.0866 (16)	0.0056 (8)	0.0125 (10)	0.0097 (10)
C8	0.0519 (10)	0.0546 (11)	0.0655 (13)	0.0047 (9)	0.0095 (9)	0.0221 (10)
C9	0.0444 (9)	0.0561 (10)	0.0393 (8)	−0.0004 (8)	0.0062 (7)	0.0092 (8)
C10	0.0398 (7)	0.0362 (7)	0.0336 (7)	−0.0002 (6)	0.0064 (6)	−0.0001 (6)
C11	0.0496 (10)	0.0624 (12)	0.0509 (11)	−0.0095 (9)	−0.0028 (8)	−0.0024 (9)
C12	0.0443 (9)	0.0565 (10)	0.0362 (8)	−0.0017 (8)	0.0059 (6)	0.0082 (7)
O3	0.1263 (17)	0.0833 (12)	0.0346 (7)	−0.0132 (11)	0.0108 (9)	0.0083 (7)
O4	0.0625 (8)	0.0499 (8)	0.0511 (8)	−0.0061 (6)	0.0045 (6)	0.0157 (6)
C13	0.0581 (12)	0.0672 (14)	0.0722 (15)	−0.0055 (10)	0.0040 (10)	0.0354 (12)
O5	0.0506 (7)	0.0567 (8)	0.0339 (6)	−0.0054 (6)	0.0068 (5)	−0.0157 (5)
C14	0.0521 (16)	0.0411 (16)	0.0413 (15)	−0.0052 (11)	0.0025 (11)	−0.0107 (13)
C15	0.0631 (19)	0.0476 (18)	0.0383 (15)	0.0020 (14)	0.0021 (12)	−0.0019 (13)
C16	0.0560 (13)	0.0798 (17)	0.112 (2)	0.0020 (12)	−0.0028 (14)	−0.0217 (17)
O6	0.0746 (11)	0.0750 (11)	0.0880 (13)	0.0021 (9)	0.0224 (10)	−0.0291 (10)
C14X	0.043 (3)	0.092 (6)	0.035 (3)	0.005 (3)	−0.003 (2)	−0.016 (3)
C15X	0.049 (3)	0.057 (4)	0.067 (4)	−0.002 (3)	0.007 (3)	−0.010 (3)

### Geometric parameters (Å, °)

**Table:**

S1—O2	1.4249 (14)	C12—O4	1.334 (3)
S1—O1	1.4285 (15)	O4—C13	1.444 (2)
S1—N2	1.6384 (14)	C13—H13A	0.9600
S1—C10	1.7484 (17)	C13—H13B	0.9600
N2—C3	1.430 (2)	C13—H13C	0.9600
N2—C11	1.478 (2)	O5—C14X	1.426 (6)
C3—C4	1.358 (2)	O5—C14	1.488 (3)
C3—C12	1.487 (2)	C14—C15	1.486 (5)
C4—O5	1.3559 (18)	C14—H14A	0.9700
C4—C5	1.470 (2)	C14—H14B	0.9700
C5—C6	1.399 (2)	C15—C16	1.413 (5)
C5—C10	1.399 (2)	C15—O6	1.465 (4)
C6—C7	1.381 (3)	C15—H15	0.9800
C6—H6	0.9300	C16—O6	1.416 (3)
C7—C8	1.387 (3)	C16—C15X	1.441 (7)
C7—H7	0.9300	C16—H16A	0.9700
C8—C9	1.389 (3)	C16—H16B	0.9700
C8—H8	0.9300	C16—H16C	0.9699
C9—C10	1.385 (2)	C16—H16D	0.9701
C9—H9	0.9300	O6—C15X	1.469 (7)
C11—H11A	0.9600	C14X—C15X	1.462 (11)
C11—H11B	0.9600	C14X—H14C	0.9700
C11—H11C	0.9600	C14X—H14D	0.9700
C12—O3	1.196 (2)	C15X—H15X	0.9800
			
O2—S1—O1	119.39 (9)	H13B—C13—H13C	109.5
O2—S1—N2	108.14 (9)	C4—O5—C14X	120.7 (3)
O1—S1—N2	107.59 (8)	C4—O5—C14	115.97 (16)
O2—S1—C10	110.29 (9)	C15—C14—O5	110.7 (3)
O1—S1—C10	109.25 (8)	C15—C14—H14A	109.5
N2—S1—C10	100.47 (7)	O5—C14—H14A	109.5
C3—N2—C11	115.86 (14)	C15—C14—H14B	109.5
C3—N2—S1	114.17 (11)	O5—C14—H14B	109.5
C11—N2—S1	117.46 (12)	H14A—C14—H14B	108.1
C4—C3—N2	119.59 (14)	C16—C15—O6	58.9 (2)
C4—C3—C12	124.33 (15)	C16—C15—C14	120.8 (4)
N2—C3—C12	116.07 (15)	O6—C15—C14	112.5 (3)
O5—C4—C3	121.52 (15)	C16—C15—H15	117.0
O5—C4—C5	116.50 (15)	O6—C15—H15	117.0
C3—C4—C5	121.65 (14)	C14—C15—H15	117.0
C6—C5—C10	117.72 (16)	C15—C16—O6	62.37 (18)
C6—C5—C4	120.88 (15)	O6—C16—C15X	61.9 (3)
C10—C5—C4	121.28 (14)	C15—C16—H16A	117.5
C7—C6—C5	120.10 (19)	O6—C16—H16A	117.5
C7—C6—H6	120.0	C15X—C16—H16A	152.0
C5—C6—H6	120.0	C15—C16—H16B	117.5
C6—C7—C8	121.39 (19)	O6—C16—H16B	117.5
C6—C7—H7	119.3	C15X—C16—H16B	86.1
C8—C7—H7	119.3	H16A—C16—H16B	114.6
C7—C8—C9	119.55 (19)	C15—C16—H16C	85.6
C7—C8—H8	120.2	O6—C16—H16C	117.6
C9—C8—H8	120.2	C15X—C16—H16C	117.5
C10—C9—C8	118.92 (18)	H16B—C16—H16C	124.8
C10—C9—H9	120.5	C15—C16—H16D	151.9
C8—C9—H9	120.5	O6—C16—H16D	117.6
C9—C10—C5	122.31 (16)	C15X—C16—H16D	117.7
C9—C10—S1	122.33 (13)	H16A—C16—H16D	88.2
C5—C10—S1	115.35 (12)	H16C—C16—H16D	114.7
N2—C11—H11A	109.5	C16—O6—C15	58.70 (19)
N2—C11—H11B	109.5	C16—O6—C15X	59.9 (3)
H11A—C11—H11B	109.5	O5—C14X—C15X	112.1 (6)
N2—C11—H11C	109.5	O5—C14X—H14C	109.2
H11A—C11—H11C	109.5	C15X—C14X—H14C	109.2
H11B—C11—H11C	109.5	O5—C14X—H14D	109.2
O3—C12—O4	123.89 (18)	C15X—C14X—H14D	109.2
O3—C12—C3	125.4 (2)	H14C—C14X—H14D	107.9
O4—C12—C3	110.75 (15)	C16—C15X—C14X	122.4 (8)
C12—O4—C13	116.76 (18)	C16—C15X—O6	58.2 (3)
O4—C13—H13A	109.5	C14X—C15X—O6	108.5 (7)
O4—C13—H13B	109.5	C16—C15X—H15X	117.4
H13A—C13—H13B	109.5	C14X—C15X—H15X	117.4
O4—C13—H13C	109.5	O6—C15X—H15X	117.4
H13A—C13—H13C	109.5		
			
O2—S1—N2—C3	−172.61 (13)	N2—S1—C10—C5	39.59 (14)
O1—S1—N2—C3	57.18 (14)	C4—C3—C12—O3	8.5 (3)
C10—S1—N2—C3	−57.04 (13)	N2—C3—C12—O3	−170.9 (2)
O2—S1—N2—C11	−32.06 (16)	C4—C3—C12—O4	−171.60 (16)
O1—S1—N2—C11	−162.27 (14)	N2—C3—C12—O4	9.0 (2)
C10—S1—N2—C11	83.50 (14)	O3—C12—O4—C13	−2.0 (3)
C11—N2—C3—C4	−101.63 (19)	C3—C12—O4—C13	178.12 (16)
S1—N2—C3—C4	39.6 (2)	C3—C4—O5—C14X	81.6 (5)
C11—N2—C3—C12	77.8 (2)	C5—C4—O5—C14X	−104.9 (5)
S1—N2—C3—C12	−141.04 (13)	C3—C4—O5—C14	126.8 (2)
N2—C3—C4—O5	177.68 (14)	C5—C4—O5—C14	−59.7 (2)
C12—C3—C4—O5	−1.7 (3)	C4—O5—C14—C15	−79.8 (3)
N2—C3—C4—C5	4.5 (2)	C14X—O5—C14—C15	27.8 (4)
C12—C3—C4—C5	−174.86 (15)	O5—C14—C15—C16	157.0 (3)
O5—C4—C5—C6	−20.4 (2)	O5—C14—C15—O6	90.8 (3)
C3—C4—C5—C6	153.18 (17)	C14—C15—C16—O6	−99.3 (3)
O5—C4—C5—C10	163.78 (14)	O6—C15—C16—C15X	79.6 (5)
C3—C4—C5—C10	−22.7 (2)	C14—C15—C16—C15X	−19.7 (5)
C10—C5—C6—C7	1.3 (3)	C15X—C16—O6—C15	−39.9 (4)
C4—C5—C6—C7	−174.71 (17)	C15—C16—O6—C15X	39.9 (4)
C5—C6—C7—C8	−0.2 (3)	C14—C15—O6—C16	113.4 (4)
C6—C7—C8—C9	−0.5 (3)	C16—C15—O6—C15X	−81.5 (5)
C7—C8—C9—C10	0.1 (3)	C14—C15—O6—C15X	31.9 (5)
C8—C9—C10—C5	1.0 (3)	C4—O5—C14X—C15X	60.1 (8)
C8—C9—C10—S1	179.64 (14)	C14—O5—C14X—C15X	−34.6 (5)
C6—C5—C10—C9	−1.7 (2)	C15—C16—C15X—C14X	11.7 (5)
C4—C5—C10—C9	174.28 (15)	O6—C16—C15X—C14X	92.8 (6)
C6—C5—C10—S1	179.57 (13)	C15—C16—C15X—O6	−81.2 (5)
C4—C5—C10—S1	−4.4 (2)	O5—C14X—C15X—C16	−164.2 (5)
O2—S1—C10—C9	−25.20 (17)	O5—C14X—C15X—O6	−100.7 (7)
O1—S1—C10—C9	107.90 (15)	C15—O6—C15X—C16	77.6 (5)
N2—S1—C10—C9	−139.14 (15)	C16—O6—C15X—C14X	−117.2 (7)
O2—S1—C10—C5	153.53 (13)	C15—O6—C15X—C14X	−39.7 (5)
O1—S1—C10—C5	−73.37 (14)		

### Hydrogen-bond geometry (Å, °)

**Table:**

*D*—H···*A*	*D*—H	H···*A*	*D*···*A*	*D*—H···*A*
C7—H7···O6^i^	0.93	2.49	3.377 (3)	158
C15—H15···O3^ii^	0.98	2.50	3.317 (4)	140

Symmetry codes: (i) −*x*+1, *y*+1/2, −*z*+3/2; (ii) −*x*+1, −*y*+1, −*z*+2.
